# Artificial dermis combined with split-thickness skin autograft in the treatment of hand thermal compression wounds: a single center case-control study

**DOI:** 10.3389/fsurg.2023.1304333

**Published:** 2023-12-22

**Authors:** Yuan Yuan, Xian Zhong, Jian Zhang, Chunming Shen, Guoxin Huang, Jianchao Zhang, Ke Wang, Ming Xu, Sheng Shao, Jun Yang, Da Qian

**Affiliations:** ^1^ Department of Burn and Plastic Surgery-Hand Surgery, Changshu Hospital Affiliated to Soochow University, Changshu No.1 People’s Hospital, Changshu, China; ^2^ Department of Evidence-Based Medicine Center, Xiangyang No.1 People’s Hospital, Hubei University of Medicine, Xiangyang, China; ^3^ Department of Plastic and Reconstructive Surgery, Shanghai Ninth People’s Hospital, Shanghai Jiao Tong University School of Medicine, Shanghai, China

**Keywords:** artificial dermis, thermal compression wound, wound repair, graft, vacuum sealing drainage technology

## Abstract

**Objective:**

To explore the clinical effect of artificial dermis combined with split-thickness skin autograft in treating hand thermal compression wounds.

**Methods:**

Forty-two patients in our hospital from January 2016 to October 2022 with thermal compression wounds were divided into two groups. The survival rate of autologous skin grafts seven days after skin grafting, the number of operations, total hospital stay, total hospitalization cost, and bacterial culture results of secretions were recorded. The visual analog scale was used to evaluate the wound pain. The condition of skin graft rupture was recorded and the scar status of the donor site was evaluated by the Vancouver Scar Scale.

**Results:**

It showed combination of artificial dermis, split-thickness skin autograft, and vacuum sealing drainage improves the treatment of hand thermal compression wounds by enhancing the survival rate of skin grafting (95.24% > 66.67%), reducing the number of operations (*P* < 0.001), relieving wound pain (*P* < 0.001), effectively controlling wound infection (4.76% < 9.52%), and reducing the skin graft rupture rate after surgery (4.8% < 28.6%). There was no evident scar hyperplasia in the donor (*P* = 0.003) and skin graft areas (*P* < 0.001), which had a good recovery of hand function (*P *= 0.037); however, this treatment strategy may prolong the hospital stay (*P *= 0.030) and increase the total hospitalization cost (*P* = 0.030).

**Conclusion:**

The composite transplantation of artificial dermis and split-thickness skin combined with the VSD significantly improves treatment and aesthetic outcomes in patients with thermal compression wounds to the hand, which is worth promoting and applying in clinical practice.

## Introduction

Various hot-pressing machines are widely used in modern manufacturing production. Due to lack of training, lack of experience, and fatigue, workers are prone to accidental injuries, with hand thermal compression injuries being the most common. Thermal compression injury is a compound heat and pressure injury characterized by a wound to a small area, severe damage, and involvement of the entire skin layer. Thermal compression is often combined with deep tissue damage, and the wounded surface can become progressively necrotic (1). Treatment of this type of injury typically involves vacuum sealing drainage (VSD) for wound preparation, followed by a medium-thickness skin graft. If combined with bone and tendon exposure, skin flap grafting is required (2). Despite being the primary treatment method, this strategy can easily cause damage to the donor area, and the appearance and function of the graft area are not satisfactory.

Previous studies (3) have reported that artificial dermal grafting can effectively reduce the difficulty and risk of surgery and produce repair effects similar to thick skin grafting. In 1980, Yannas and Burke developed the first membrane artificial skin synthetic material, Integra (4), and the Japanese GENZE Corporation further improved and optimized the basis of Integra and developed Pelnac (5). Additionally, the Lando® double layer artificial dermal repair material produced by Shenzhen Qikang Medical Instrument Co., Ltd. has a similar structure to Integra artificial synthetic dermal material from the United States. The upper layer is silicone, which controls water loss and blocks external bacteria. The lower layer is made from a collagen sponge, which provides a good scaffold, pore structure, and mechanical properties that can promote the proliferation of fibroblasts and capillary neovascularization. Together, these factors facilitate wound preparation of dermal grafting with abundant blood supply (6).

The application of artificial dermis in treating various skin and soft tissue defects has received extensive attention (7), especially in treating exposed bone and tendon wounds, in which this treatment strategy has achieved good efficacy (8–10). However, few reports have assessed applying artificial dermis combined with split-thickness skin autograft in treating hand thermal compression wounds. This article analyzed and compared the clinical effects of artificial dermis combined with split-thickness skin autograft and simple medium-thickness skin autograft in treating hand thermal compression wounds. Additionally, the clinical application value of artificial dermis and split-thickness skin autograft followed by VSD in treating hand thermal compression wounds was comprehensively evaluated, providing a reference for the clinical management of hand thermal compression wounds.

## Subjects and methods

### Ethics

The hospital's Medical Ethics Committee approved this study, and all patients and their families were fully informed of the treatment plan and possible risks and voluntarily signed informed consent.

#### Inclusion criteria

(1) Aged between 18 and 65 years, were admitted to the hospital within 24 h after injury, were diagnosed with third-degree burns on the hand, whose injury site was unilateral, the cause of injury was thermal compression, and the first operation was performed within three days after admission; (2) During the stage I, artificial dermis combined with VSD treatment was used, followed by split-thickness skin autograft grafting in the stage II, or medium-thickness skin autograft grafting in the stage II after VSD treatment in the stage I; (3) The size of the wound was less than 60 cm^2^ and the injured hand was not complicated with fracture; (4) The patient or their family members voluntarily signed an informed consent form for the treatment and surgical plan; (5) The relevant clinical data was complete.

#### Exclusion criteria

**Figure 1:**
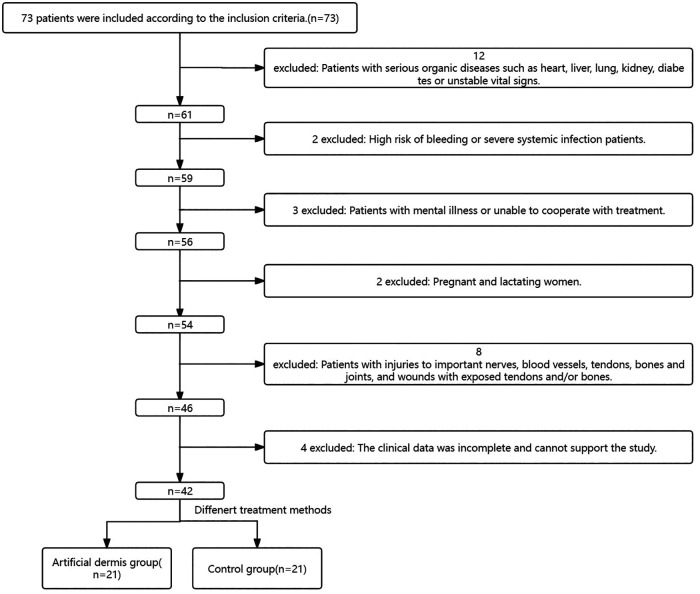
Inclusion and exclusion criteria screening flow chart.

(1) Patients with serious organic diseases such as heart, liver, lung, kidney, diabetes or unstable vital signs; (2) High risk of bleeding or severe systemic infection patients; (3) Patients with mental illness or unable to cooperate with treatment; (4) Pregnant and lactating women; (5) Patients with injuries to important nerves, blood vessels, tendons, bones and joints, and wounds with exposed tendons and/or bones; (6) The clinical data was incomplete and cannot support the study (Figure 1).

### Clinical data

The clinical data of 42 cases of continuously enrolled patients with hand thermal compression injuries admitted to the Department of Burn and Plastic Surgery of The First People's Hospital of Changshu City from January 2016 to October 2022 were retrospectively analyzed. The patients were divided into an artificial dermis group and a control group according to treatment methods. The control group was treated with VSD at stage Ⅰ and received medium-thickness skin autograft at stage Ⅱ while the artificial dermis group was treated with artificial dermis combined with VSD at stage Ⅰ and received split-thickness skin autograft at stage Ⅱ.

There were 21 patients in the artificial dermis group, including 13 males and 8 females, aged 21–53 years, with an average age of 37.81 ± 8.60 years, a wound size range of 6–44 cm^2^, and a median wound size of 10.00 (9.00–15.00) cm^2^. Eschar formed on the wound, involving the entire skin layer, and the depth of all wounds was characterized as third-degree. The control group was treated with VSD at stage Ⅰ and received medium-thickness skin autograft at stage Ⅱ. In the control group, there were 21 patients, including 11 males and 10 females, aged 24–49 years, with an average age of 36.67 ± 6.87 years. The wound size was 4.5–27 cm^2^, and the median wound size was 11.00 (8.00–15.00) cm^2^. Eschar formed on the wound, involving the entire skin layer, and the depth of all wounds was determined as third-degree. There were no statistical differences in gender, age, and wound size between the two groups (*P* > 0.05), which were comparable (Table 1).

**Table 1:** Demographic and clinical characteristics of patients in artificial dermis and control groups.

Variables	Control group (*N* = 21)	Artificial dermis group (*N* = 21)	*P*-value
Gender, *n* (%)			
Female	10 (47.62%)	8 (38.10%)	0.755
Male	11 (52.38%)	13 (61.90%))	
Age, Mean ± SD, years	36.67 ± 6.87	37.81 ± 8.60	0.637
Wound size, median (IQR), cm^2^	11.00 (8.00–15.00)	10.00 (9.00–15.00)	0.840
The number of operations, mean (SD)			<0.001
2	8 (38.10%)	20 (95.24%)	
3	12 (57.14%)	1 (4.76%)	
4	1 (4.76%)	0 (0%)	
Total hospital stay, mean (SD), days	24.00 ± 4.24	26.67 ± 3.38	0.030
Total hospitalization costs, mean (SD), CNY	26171.86 ± 5758.19	33981.30 ± 9117.39	0.002
TAM, mean (SD), score	48.19 ± 2.93	50.19 ± 3.08	0.037
VSS for graft area, median (IQR), score	3.00 (3.00–4.00)	3.00 (2.00–3.00)	0.005
VSS for donor area, median (IQR), score	8.00 (8.00–9.00)	1.00 (1.00–1.00)	<0.001
VAS before operation, median (IQR), score	3.00 (3.00–5.00)	3.00 (2.00–4.00)	0.451
VAS after 7 days of first operation, median (IQR), score	2.00 (2.00–3.00)	1.00 (0.00–1.00)	<0.001
Skin graft survival status, *n* (%)			0.060
Necrosis	1 (4.76%)	0 (0%)	
Partial survival	6 (28.57%)	1 (4.76%)	
Complete survival	14 (66.67%)	20 (95.24%)	
Skin rupture status, *n* (%)			0.098
Negative	15 (71.43%)	20 (95.24%)	
Positive	6 (28.57%)	1 (4.76%)	
Bacterial culture results before operation, *n* (%)			1.000
Negative	19 (90.48%)	20 (95.24%)	
Positive	2 (9.52%)	1 (4.76%)	
Bacterial culture results 7 days after first operation, *n* (%)			1.000
Negative	20 (95.24%)	20 (95.24%)	
Positive	1 (4.76%)	1 (4.76%)	

CNY, ChineseYuan; TAM, total active motion; VSS, vancouver scar scale; VAS, visual analog scale.

### Treatment methods

#### Preoperative preparation

After admission, all patients were disinfected with 10% iodophor, bandaged with iodophor gauze, empirically treated with antibiotics for infection prevention, received symptomatic analgesia and detumescence treatment, and had their dressings changed daily. Before surgery, wound secretions were taken for bacterial culture and drug sensitivity testing. Anti-infection treatment was performed according to the test results. Chest CT, electrocardiogram, routine blood routine, hypersensitive C-reactive protein, blood biochemistry, coagulation function, and other examinations were completed in all patients, and preoperative preparation was actively conducted to exclude surgical contraindications.

### Surgical methods

#### The artificial dermis group

The artificial dermis group was treated with artificial dermis combined with VSD at stage Ⅰ and split-thickness skin autograft at stage Ⅱ. After the induction of anesthesia, the surgical position was selected, the tourniquet was tied at the middle and upper 1/3 of the upper arm, disinfection and dressing were performed, and the tissue was disinfected and spread. Next, the denatured and necrotic tissue was removed from the deep fascia layer, and the parabiotic tissue was preserved. Any bleeding was stopped by bipolar electrocoagulation. The wound was then washed with 10% iodophor and 0.9% normal saline three times each, the tourniquet was released, and the bleeding was stopped again. Next, the Lando® double layer artificial dermis (Shenzhen Qikang Medical Equipment Co., LTD.) was soaked in normal saline for 15–20 min, and the artificial dermis was transplanted onto the wound surface after debridement, with the silicone layer facing up and the collagen sponge layer attached to the wound. The wound was trimmed to a suitable size and then fixed with a silk suture. A pointed blade was used to puncture the silicone layer of the artificial dermis to promote drainage. Medical foam dressing of appropriate size (1 cm beyond the edge of the artificial dermis graft area as the standard) (Wuhan VSD Medical Science & Technology Co., LTD.) covered the artificial dermis graft area and was fixed in place. Then, the dressed area was closed with a semi-permeable film, connected to each drainage tube, and connected to the negative pressure device. A comprehensive inspection was carried out to ensure that the medical foam dressing was dented, the tube shape appeared, and there was no air leakage, proving that the negative pressure device was in good working condition and the negative pressure was adequate. After surgery, continuous negative pressure drainage was given at −125 mmHg, and anti-inflammatory and symptomatic treatments were administered. During this period, careful observation was needed to ensure the pipeline was unobstructed and assess whether fluid drainage was abnormal. The VSD material was removed 5–7 days later. After that, the dressing was changed every two days, and the formation of dermal tissue was observed. When the color of the artificial dermis changed to golden yellow and the surface silicone film began to separate from the underlying dermoid tissue, the silicone film was peeled off, and the sticky exudates on the surface of the collagen layer were scraped off. According to the size of the wound, the split-thickness skin of the outer edge of the thigh was transplanted into the wound and fixed by suture. The donor and graft areas were bound with appropriate pressure, and anti-inflammatory and symptomatic treatment were performed after surgery. The condition of the skin graft was examined 5–7 days later.

#### Control group

The control group was treated with VSD at stage Ⅰ and received medium-thickness skin autograft at stage Ⅱ. The specific operation methods and postoperative treatment of debridement and VSD were the same as those of the artificial dermis group. After the first VSD treatment, if the wound preparation did not meet the requirements for skin autograft, debridement, and VSD was performed again until the wound was clean, no bone and tendon was exposed, and the granulation growth was satisfactory (the granulation tissue was bright red and granular, the lacunar was filled with fresh granulation tissue, there was no edema or excessive proliferation of granulation tissue, and the wound surface was free of infectious secretions or pus). Then, medium-thickness skin autograft was performed.

Both groups started active or passive functional rehabilitation training after the wound healed to prevent joint stiffness. At the same time, silicone therapy combined with pressure therapy was performed by all patients to prevent and treat scar hyperplasia.

### Observation indicators

The survival rate of autologous skin grafts seven days after skin grafting was evaluated using the following formula: Survival rate of skin grafting = the number of cases of complete survival after skin grafting/total number of patients × 100%. The criteria for complete survival of skin grafting were adequate adhesion between the skin graft and the wound bed, with the growth of new blood vessels, and no necrosis or detachment of the skin graft. Additionally, the number of operations, total hospital stay, total hospitalization cost, pain scores of wounds before and seven days after treatment, and bacterial culture results of wound secretions were recorded and compared. The time point prior to treatment was defined as data recorded before the first debridement. Conversely, post-treatment data were defined as data recorded after the first debridement. Bacterial infection was assessed with the following formula: The positive rate of bacterial culture in secretions = number of positive cases of bacterial culture/total number of patients × 100%. Patient pain was evaluated using the Visual Analog Scale (VAS) (11); that was, a vernier slide caliper with a 0–10 cm scale was used, and patients were asked to move the scale to the corresponding position according to their feelings, with a range of 0–10 points. The degree of pain was positively correlated with the score, i.e., where 10 would reflect extreme pain. All patients were followed up after autologous skin grafts to observe whether there was a rupture within six months and calculate the rupture rate of the skin grafts. Skin rupture was assessed using the following formula: The rupture rate of the skin grafts = the number of cases of skin grafts rupture/the total number of patients × 100%. Six months after skin grafting, scar scores were measured on the graft and donor areas using the Vancouver Scar Scale (VSS) (12). Parameters such as color, vascular distribution, thickness, and softness were scored, ranging from 0 to 15 points. The higher the score, the heavier the scar. TAM (Total Active Motion), recommended by the Chinese Medical Association Hand Surgery Society, was used to evaluate the recovery of hand function after six months of the operation (8). Two department physicians with attending titles or higher independently evaluated the patient's scars according to the VSS scoring table. The average value was recorded.

### Statistics

SPSS 26.0 and RStudio (R version 4.2.1) was used to analyze the data. Discontinuous variables were presented as percentages while continuous variables in normal distribution were described as mean ± standard deviation (SD) or else reported as median (Range). Measurement data such as operation times, total hospital stay, total hospitalization cost, VAS pain score, VSS score of the donor and graft area, and TAM score followed a normal distribution and were expressed as a mean ± standard deviation $\left(\bar{x}\pm s\right)$. The independent sample *t*-test was used for comparison between groups. Measurement data that did not follow the normal distribution were described by the median (upper quartile, lower quartile) and compared using non-parametric tests. Count data such as survival rate of skin grafting, bacterial culture skin grafting, and skin rupture were expressed as frequency (%), and the χ^2^ test was used for comparison (Chi-square value was not output when Fisher's exact probability method was used). The “autoReg” package was used to make the Baseline table. The “ggplot2”, “ggsignif”, “ggprism” packages were used for drawing figures. *P* < 0.05 indicated that the difference was statistically significant.

## Results

### Patients-groups

A total of 42 patients were included in this study, of which 21 were treated with artificial dermis combined with VSD at stage Ⅰ and split-thickness skin autograft at stage Ⅱ and were included in the artificial dermis group; 21 patients were treated with VSD at stage Ⅰ and received medium-thickness skin autograft at stage Ⅱ and were included in the control group. The related information was showed in Table 1.

### Survival rate of skin grafting

**Figure 2:**
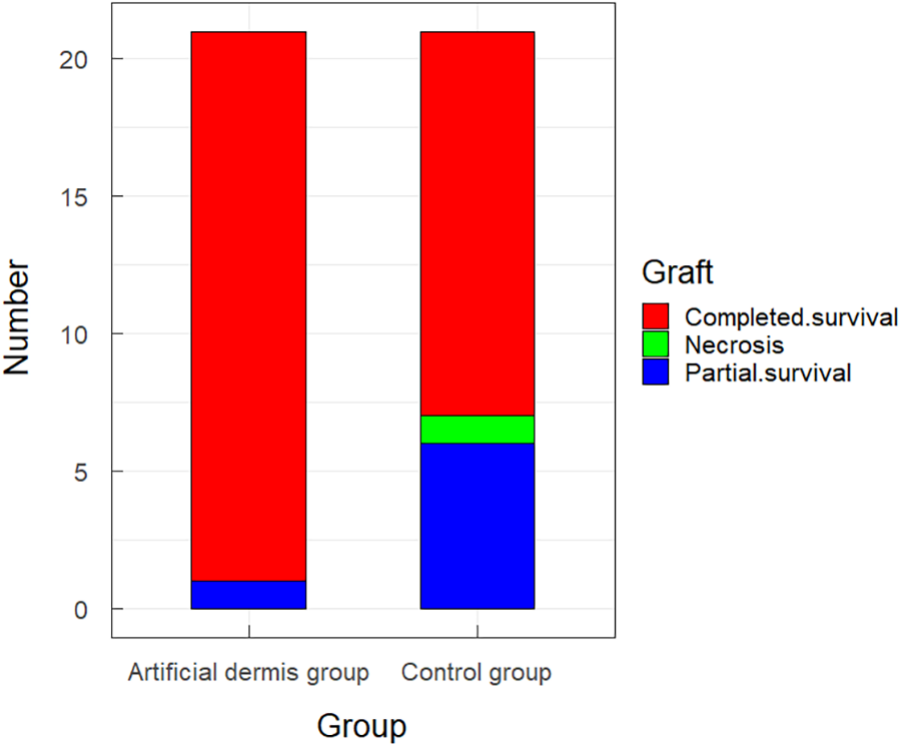
The survival rate of skin grafting in the artificial dermis group and in the control group.

Seven days after autologous skin grafting, 20 of the 21 (95.24%) patients in the artificial dermis group had complete graft survival, and one had local poor graft survival, which healed after a dressing change. Among the 21 patients in the control group, 14 cases (66.67%) had complete graft survival, 6 had poor local graft survival and were healed after dressing change, and 1 had necrosis and healed after receiving an additional skin graft. The survival rate of skin grafting in the artificial dermis group was significantly higher than in the control group (Figure 2).

### Postoperative wound conditions of patients

**Figure 3:**
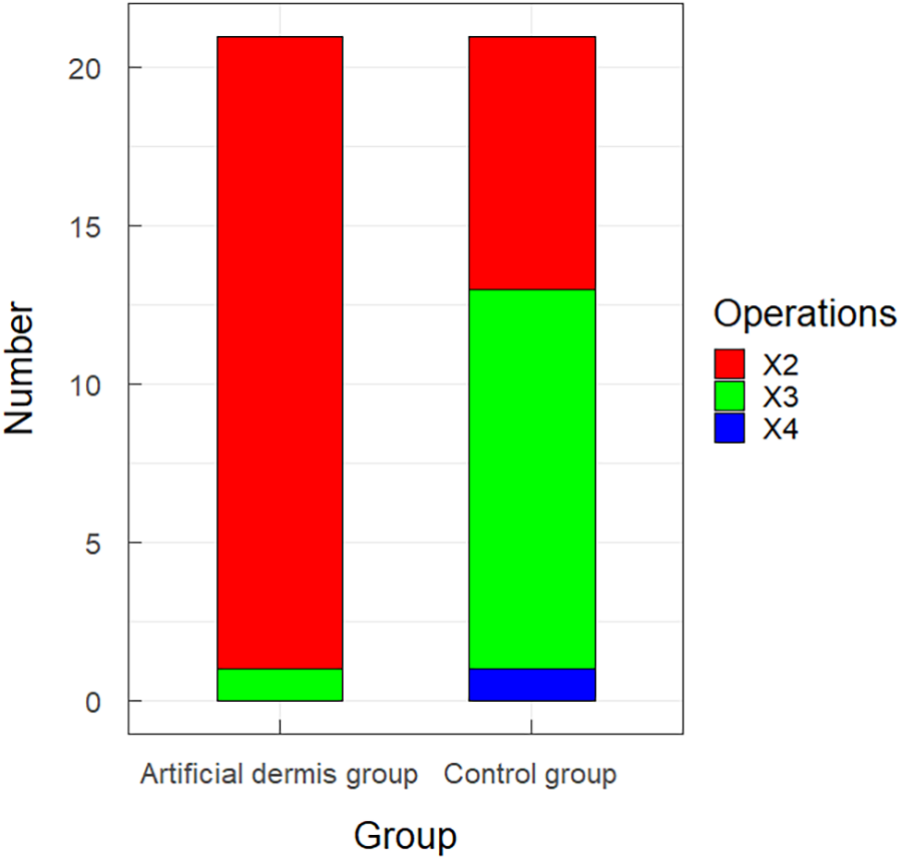
The number of surgeries performed two, three or four times in the artificial dermis group in the control group.

**Figure 4:**
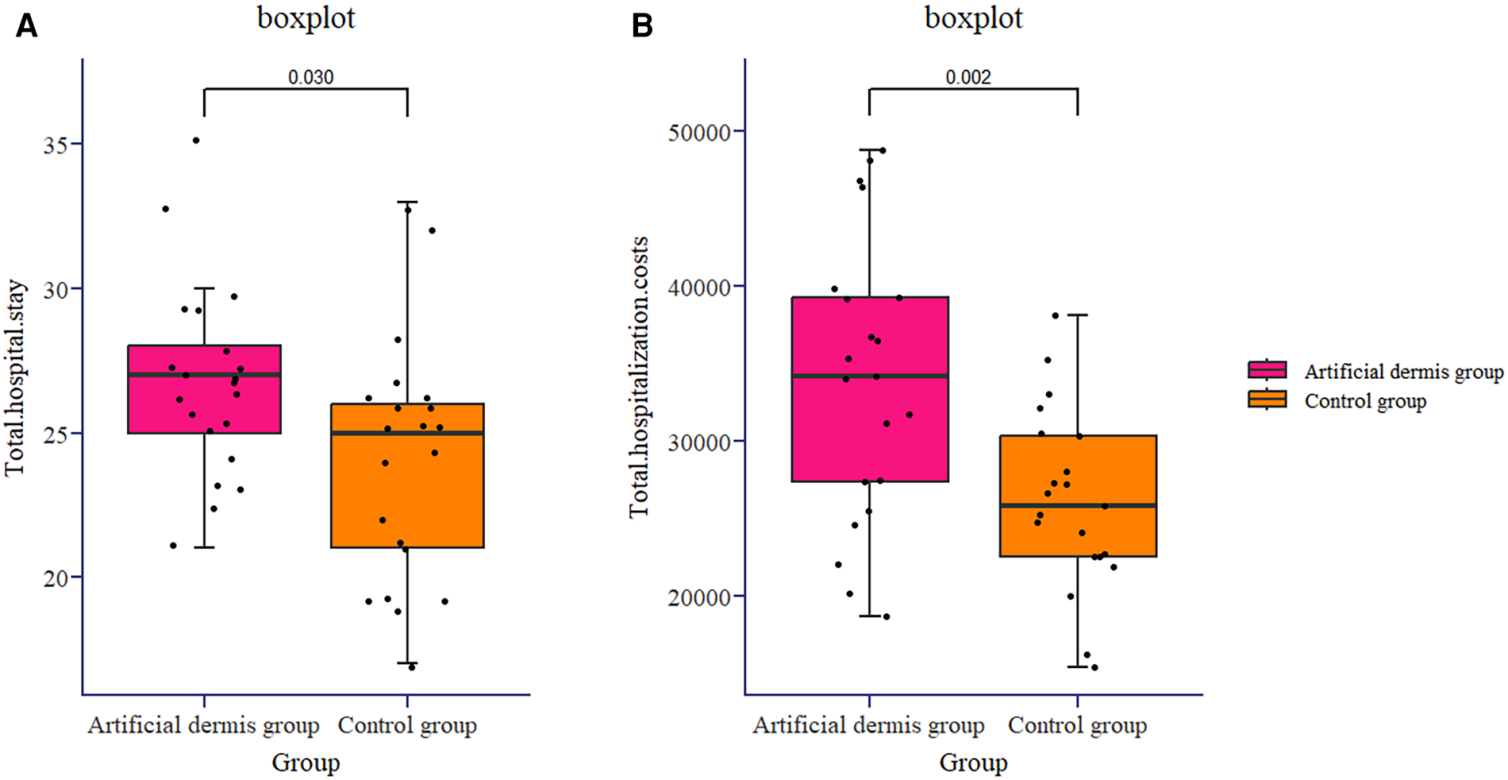
The total hospital stay of the artificial dermis group and the control group [(**A**), (*P *= 0.030)]. The total hospitalization cost of the artificial dermis group and the control group [(**B**), (*P* = 0.002)].

Next, the number of operations, total hospital stay, total hospitalization cost, wound pain score before treatment and seven days after treatment, and positive rate of wound secretion culture before treatment and seven days after treatment were compared between the two groups. The number of surgeries performed three or four times in the artificial dermis group was only one, significantly less than that in the control group (Figure 3). The total hospital stay of the artificial dermis group was 26.67 ± 3.38 days, significantly longer than 24.00 ± 4.24 days in the control group (Figure 4A). The total hospitalization cost of the artificial dermis group was 33,981.30 ± 9,117.39 yuan, significantly higher than 26,171.86 ± 5,758.19 yuan in the control group (Figure 4B). The pain scores of wounds of the two groups were similar before treatment, while the pain score of wounds of the artificial dermis group was significantly lower after treatment (Table 1).

The number of positive cases of bacterial culture in wound secretions before and after treatment in the artificial dermis and control groups was similar, with one case (4.76%) being observed in the artificial dermis group, while the number of positive cases before and after treatment in the control group was 2 cases (9.52%) and 1 case (4.76%), respectively.

### Follow up on the condition of graft rupture, scar formation and hand function

**Figure 5:**
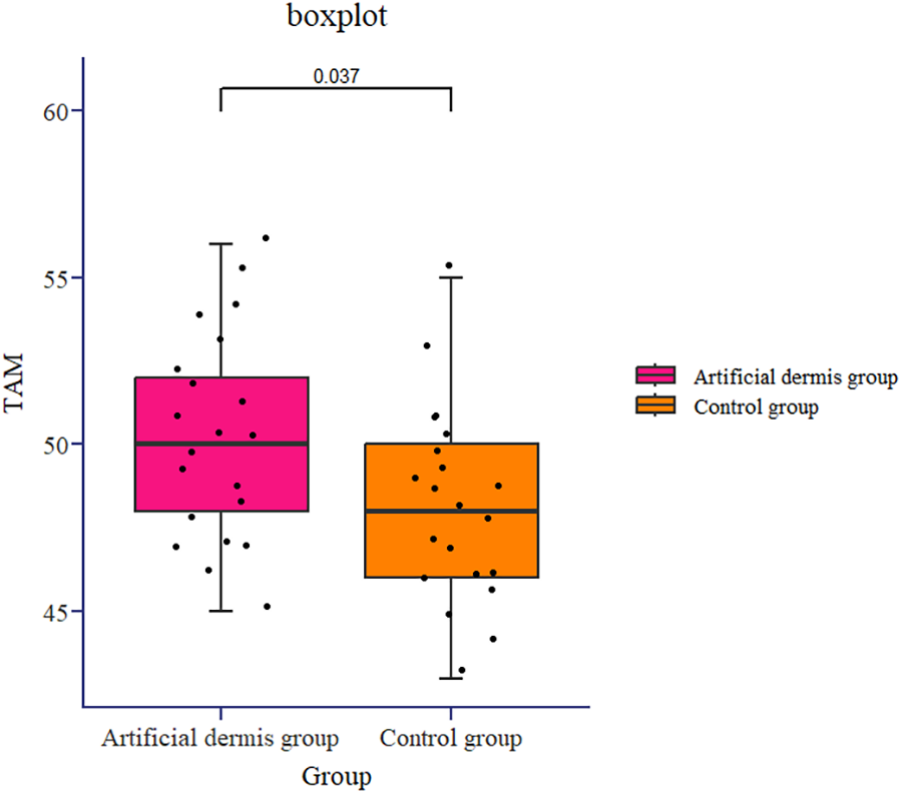
The TAM score of hand function of patients in artificial dermis group and in control group (*P *= 0.037). TAM: Total Active Motion.

Within six months after skin grafting, one case (4.76%) in the artificial dermis group and 6 cases (28.57%) in the control group experienced skin graft rupture, indicating significantly less graft rupture in the artificial group. Additionally, the VSS scores of the skin graft and the skin donor area in the artificial dermis group were lower than those in the control group (both *P* < 0.05), with the VSS scores of the skin graft area being 3.00 (2.00, 3.00) and the skin donor area being 1.00 (1.00, 1.00) in the artificial dermis group, and 3.00 (3.00, 4.00) and 8.00 (8.00, 9.00) in the control group, respectively (Table 1). Moreover, the TAM score of hand function of patients in artificial dermis group was 50.19 ± 3.08, which were significantly higher than 48.19 ± 2.93 of control group (Figure 5, *P* = 0.037).

### Description of a typical case

**Figure 6:**
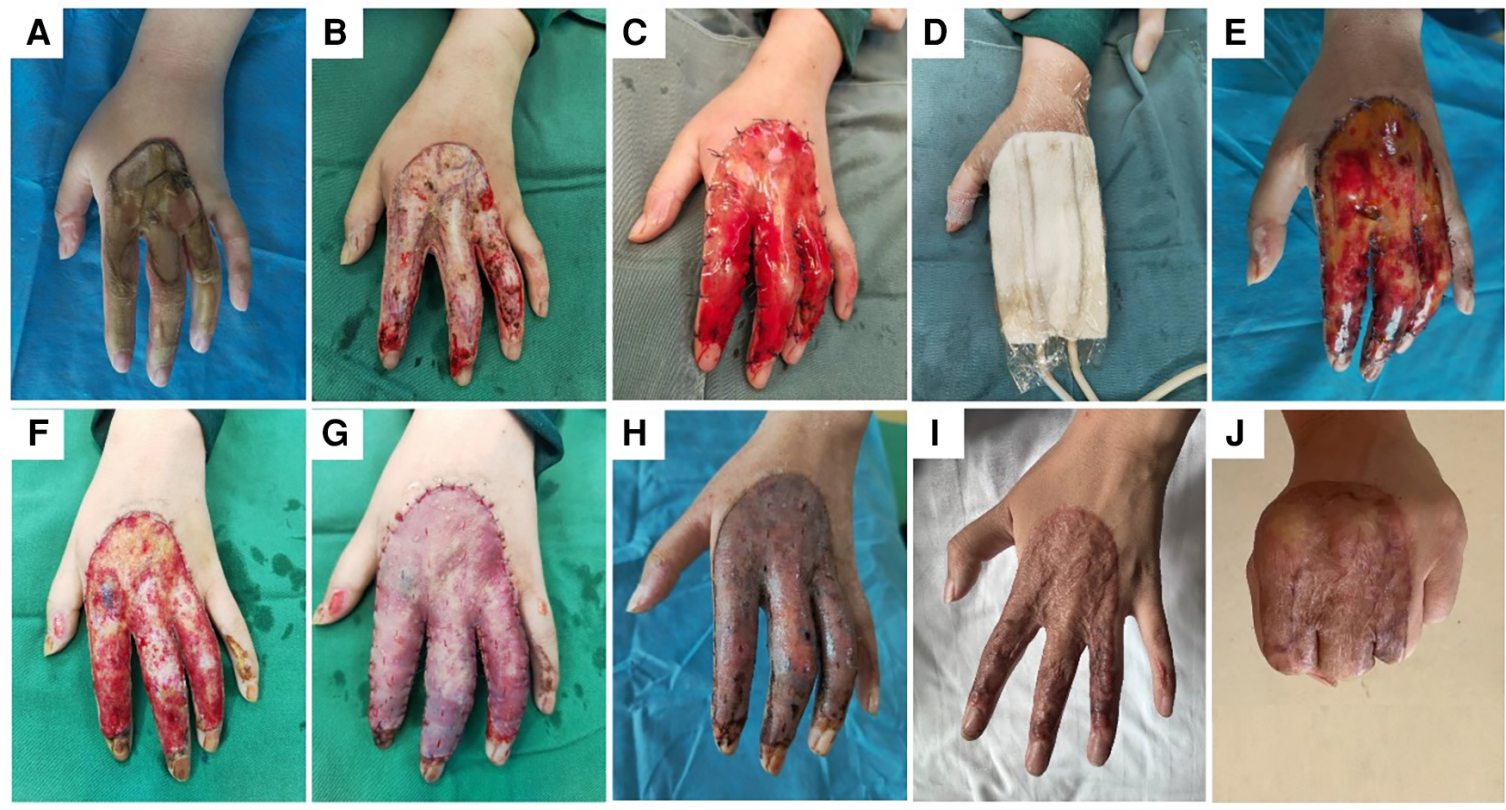
Composite transplantation of artificial dermis and split-thickness skin combined with VSD for repairing hand thermal compression wounds. (**A**) Preoperative wound condition; (**B**) after the first debridement and removal of the scab, the blood flow of the wound bed was general, and most of the tissues were parabiotic; (**C**,**D**) immediately after artificial dermis grafting and VSD; (**E**,**F**) 17 days after artificial dermis grafting, the vascularization of artificial dermis was satisfactory; (**G**) immediately after split-thickness skin autograft; (**H**) 7 days after split-thickness skin autograft; (**I**). (**J**) Follow up for six months after split-thickness skin autograft.

A 21-year-old female was admitted to the hospital due to a heat machine pressure injury on the left hand, which remained extremely painful for 1 h. The patient was diagnosed with a thermal compression injury with third-degree burns (1%) on the left hand. Specialized examination showed thermal compression wounds on the back of the left hand and the back of the fingers, among which flaky third-degree wounds about 11 cm*4 cm on the distal fingers of the second, third, and fourth fingers extended to the back of the hand. The back of the first and fifth fingers each showed a third-degree wound with a pale base and no tenderness. Noticeable swelling of the left hand was observed. On the third day of admission, the patient underwent scab resection, artificial dermis grafting, and VSD under general anesthesia. The wound negative pressure material was removed on the seventh day after surgery, and it was found that the artificial dermis was well fitted to the wound bed, without subcutaneous fluid accumulation or abnormal secretions. The wound dressing was changed once every two days, and vascularization of the artificial dermis was observed. On the 17th day after the artificial dermis grafting, the vascularization of the artificial dermis was satisfactory, and a split-thickness skin autograft was performed under general anesthesia. On the seventh day after split-thickness skin autograft, the dressing in the skin graft area was opened, and the grafted skin looked satisfactory. Follow-up after discharge showed good survival of skin grafts, no rupture, good mixed color, good texture, no cicatricial contracture, good range of motion, and no functional impact (Figure 6).

## Discussion

The hand is an important functional organ of the human body; however, the skin of the hand is thin and delicate in structure, making it sensitive to aesthetic changes. Thermal compression injury of the hand is a common emergency in the burn and plastic surgery department. Dysfunction and decreased aesthetics after healing are common if the repair method is not appropriately selected. To repair such wounds, traditional surgery requires thorough debridement (13), which inevitably leads to premature exposure of deep tissue and requires transplantation of free or adjacent flaps. It is well understood that this kind of surgery is complicated, with limited flap selection that often significantly damages the donor area. Furthermore, after healing, the wound is prone to skin flap swelling and scar hyperplasia, which affects function and appearance (14).

In clinical practice, VSD and medium-thickness skin grafts are commonly used and mature techniques. VSD is commonly used in the wound preparation stage to cultivate a wound bed with fresh granulation and rich blood supply, which is the key to skin graft survival. He S (15) reported that early tissue swelling after thermal compression injury would lead to ischemia and that it is difficult to accurately define deep tissue stasis and necrosis zones. During debridement, the parabiotic tissue should be preserved as much as possible, and VSD should be used to absorb abnormal exudates, reduce cell edema, accelerate cell metabolism, increase tissue perfusion volume, promote angiogenesis, and simultaneously protect and restore the parabiotic tissue. A healthy skin graft wound bed is cultivated, and a medium-thickness skin graft is used to repair the wound. This type of surgery often requires more than 2 rounds of debridement and VSD treatment. Even after VSD treatment, some wounds may exhibit local deep tissue exposure and irregular wound bed characteristics. These are all adverse factors that affect the survival of skin grafting. If skin grafting fails, treatment time is prolonged, and early functional exercise is negatively affected (16). After healing, due to factors such as skin rupture and contraction, tendon adhesion, and scar contracture, the function and appearance of the hand are often affected, and the long-term aesthetic effect is not ideal (17). With improved living standards and the need to restore hand function, people's expectations for the aesthetic as well as functional recovery of their hands after injury are also increasing.

Artificial dermis is a collagen-covering material with structural properties that can biologically mimic the microenvironment required for wound repair. Artificial dermis can induce cell adhesion, chemotaxis, and proliferation, providing nutrition and a barrier function for the wound (18). Previous work has shown that using artificial dermis in early wound coverage can effectively prevent secondary and progressive tissue damage and is conducive to the recovery of parabiotic tissues (19). Additionally, as a scaffold material, the artificial dermis can promote fibroblast proliferation capillary neovascularization, induce its growth into the pores of the inner layer collagen sponge, and promote the secretion of various extracellular matrices such as fibronectin, collagen, and laminin, improve the wound blood supply of wound bed, accelerate fresh granulation formation, and construct a skin graft wound bed which contains dermoid tissue.

Several studies have shown that the composite transplantation of artificial dermis and split-thickness skin based on VSD treatment induces less surgical trauma and facilitates a functional appearance close to normal after healing (20). After artificial dermis grafting, VSD can effectively fit the artificial dermis to the wound, promote the vascularization of the artificial dermis, and improve the treatment effect of the wound (21). Morykwa et al. (22) found that a negative pressure of −125 mmHg can optimize local tissue blood supply, thereby inducing the migration of fibroblasts and vascular endothelial cells to the dermal space. After artificial dermis vascularization, covering the exposed deep tissue can also effectively improve the abnormal growth of granulation on the wound surface and provide a smooth wound bed with good blood supply for autologous skin grafting (9, 23–25). The results of this study showed that the survival rate of skin grafting in the artificial dermis group was significantly higher than that in the control group. Furthermore, the number of additional operations was less than that in the control group, indicating that the composite transplantation of artificial dermis and split-thickness skin combined with VSD could improve the survival rate of skin grafting and reduce the need for further surgical intervention.

VSD treatment can effectively control wound infection (21). Furthermore, while artificial dermis functions as a graft without inducing immune rejection, it still possesses limited capacity for combating infections (26). In this study, the presence of bacteria in wound secretions before and after treatment was similar between the two groups, indicating that the combined use of artificial dermis combined with VSD did not affect the bacteriostatic effect of VSD. Interestingly, after treatment, the artificial dermis group had lower wound pain scores, indicating that the artificial dermis alleviated wound pain. Possible reasons for this observation could be that the artificial dermis covered the early wound, promoting the formation of dermoid tissue and accelerating the wound repair, thus reducing the pain caused by the persistence of the wound. Additionally, the combination of VSD can improve wound edema, alleviate vascular compression, promote capillary dilation, optimize local blood supply, inhibit inflammatory reactions and pain factor formation, and thus improve pain.

More attention is paid to trauma aesthetics due to the development of sophisticated and effective medical treatment strategies and aesthetics-improving surgery. It is now common for patients and clinicians to aim to improve the overall aesthetics of treatment. As such, the academic community is committed to exploring intact skin graft treatment and mild scars in the donor and graft areas (27). At the 6 month follow-up, the VSS scores of the skin graft and donor areas in the artificial dermis group were 3.00 (2.00, 3.00) and 1.00 (1.00, 1.50), respectively, which were lower than 3.00 (3.00, 4.00) and 8.00 (7.50, 9.00) of the control group, respectively. These data suggest that the artificial dermis group effectively reduces the appearance of scarring in the donor and graft areas. Skin rupture is an important indicator reflecting the long-term efficacy of wounds and an important factor leading to postoperative scar formation and aggravation. Most wounds exhibit thin skin, low subcutaneous tissue content, and repeated rupture after skin grafting (28). Therefore, seeking effective treatment methods to avoid repeated skin ruptures is the key to improving the efficacy of wound healing. Previous studies have confirmed that artificial dermis reduces postoperative skin rupture and scar hyperplasia in the surgical area (29–31). In this study, within six months of follow-up, the rate of skin rupture in the artificial dermis group was 4.76%, significantly lower than the 28.57% observed in the control group, indicating that the artificial dermis group has a greater advantage in preventing skin rupture after treatment. This observation could be due to several reasons. First, VSD can protect the ecological tissue between wounds, promote wound drainage, control wound infection, improve wound blood supply, accelerate artificial dermis vascularization, reduce the risk of scar formation, and improve the quality of wound healing (32). Second, the double-layer structure of artificial dermis can protect the wound surface and promote the regeneration of dermoid tissue in a short period. Because of these factors, the reconstruction process of this dermis is similar to the healing process of autologous skin and is similar to normal skin tissue in terms of softness, color, and other aspects. Additionally, the dermis is not prone to skin rupture, and the degree of scarring is often mild (33). The combination of VSD and artificial dermis can result in complementary advantages, and the split-thickness skin used for transplantation can reduce skin damage in the donor area, significantly reducing scar formation after healing. Di et al. (8) applied artificial dermis combined with split-thickness skin to repair exposed wounds of bones and tendons of the hands and feet in 41 cases. After surgery, all skin grafts survived, with reduced surgical complications, fewer postoperative scars, and significant functional improvement; however, wound healing and hospital stay were prolonged. In this study, the hospital stay of the artificial dermis group was also significantly longer than that of the control group, possibly because it takes about two to three weeks to generate dermoid tissue after artificial dermis grafting (10). In contrast, some wounds in the control group received medium-thickness skin grafts after receiving 2–3 VSD treatments, thus reducing the hospital stay. The extension of hospital stay will inevitably increase the cost of treatment, and the artificial dermis itself is expensive, leading to the total hospitalization cost of the artificial dermis group in this study being higher than that of the control group.

In summary, the composite transplantation of artificial dermis and split-thickness skin combined with the VSD significantly improves treatment and aesthetic outcomes in patients with thermal compression wounds to the hand. This combined strategy can improve the survival rate of skin grafting, reduce the number of operations, alleviate wound pain, effectively control wound infection, and reduce the rate of skin rupture in the postoperative skin graft area. Furthermore, there is no apparent scar hyperplasia in the donor and graft areas; however, this treatment strategy results in extended hospital stays and increases the total hospitalization cost. Despite these disadvantages, artificial dermis grafting is easy to operate, protects parabiotic tissues, reduces the exposure of bone and tendon injuries, and achieves better clinical efficacy with less surgical trauma, indicating that it is a superior technique for treating hand thermal compression injuries and is worth promoting and applying in clinical practice.

## Data Availability

The raw data supporting the conclusions of this article will be made available by the authors, without undue reservation.

## References

[1] Lu KH Luo JH Zhong DC. Clinical types and treatment of hand thermal compression injuries. J Fourth Mil Med Univ. (1984) 22(3):199–200.

[2] Dai XY Xing SL Wang YC Shen ZL Jia WX Huang XQ Application of sequential-therapy for heat-press injury of the hand. J Tissue Eng Reconstr Surg. (2011) 7(05):274–6. 10.3969/j.issn.1673-0364.2011.05.009

[3] Liu JT Zeng C Ouyang RL Huang SR. Clinical application of artificial dermis combined with vacuum sealing drainage technique and split-thickness skin autograft for repairing complex wounds. Chin J Damage Repair (Electronic Edition). (2020) 15(03):215–8. 10.3877/cma.j.issn.1673-9450.2020.03.013

[4] Monjanel B Baillif S Lagier J Gastaud L Poissonnet G Martel A. Efficacy and safety of an artificial dermal graft for the reconstruction of exenterated sockets: a preliminary report. Graefes Arch Clin Exp Ophthalmol. (2021) 259(9):2827–35. 10.1007/s00417-021-05155-7 33770270

[5] Murphy C Atkin L Dissemond J Hurlow J Tan YK Apelqvist J Defying hard-to-heal wounds with an early antibiofilm intervention strategy: ‘wound hygiene’. J Wound Care. (2019) 28(12):818–22. 10.12968/jowc.2019.28.12.818 31825771

[6] Liu K Wang YM Qi XM Sun YC Tian LJ Zhao YB Comparison of artificial dermis Lando (R) and Pelnac (R) combined with induced membrane technique in treatment of composite trauma in rabbit bilateral femurs. Chin J Orthop Trauma. (2019) 21(8):699–705. 10.3760/cma.j.issn.1671-7600.2019.08.010

[7] Dai C Shih S Khachemoune A. Skin substitutes for acute and chronic wound healing: an updated review. J Dermatolog Treat. (2020) 31(6):639–48. 10.1080/09546634.2018.1530443 30265595

[8] Di HP M XL S JJ Xue JD Liu L Guo HN A prospective randomized controlled study of the effectiveness of artificial dermis combined with split-thickness skin for repairing wounds with bone and tendon exposure in hands and feet. Chin J Burns. (2021) 37(12):1130–6. 10.3760/cma.j.cn501120-20210325-00103 PMC11917237 34839603

[9] Dong QQ Xie SQ Wang LJ Cui ZT Wu JQ Ge ZF Clinical study of vacuum sealing drainage combined with Lando artificial dermis combined with autologous skin grafting for the treatment of wounds with bone and tendon exposure and bone fracture of hand and foot. Chin J Damage Repair (Electronic Edition). (2020) 15(01):51–5. 10.3877/cma.j.issn.1673-9450.2020.01.008

[10] Gong C Tang HT Wang GY Shi SJ Wang JH Fan KW Evaluate the clinical effectiveness of domestic double layer artificial dermis dressing with autologous split-thickness skin for treatment of wounds with bone or tendon exposure. Chin J Damage Repair (Electronic Edition). (2016) 11(01):34–9. 10.3877/cma.j.issn.1673-9450.2016.01.008

[11] Gao WL Wang XH. Progress in research on visual analogue pain scoring. J Med Res. (2013) 42(12):144–6. doi: CNKI:SUN:YXYZ.0.2013-12-048

[12] Liu HB Tang D Cao HY Li KC. Reliability of Vancouver scar scale. Chin J Rehab Med. (2006) 21(3):240–2. 10.3969/j.issn.1001-1242.2006.03.015

[13] Yu JC Peng WY Chen BQ Chen GB Liu XS. Clinical analysis of patients with hot-press injury in the hands managed by auto-skin grafting with PELNAC. Chin J Damage Repair (Electronic Edition). (2008) 04:454–7. 10.3969/j.issn.1673-9450.2008.04.010

[14] Wang J Jing G Dong DQ Pan YC. Artificial dermis combined with autologous thin skin graft for repairing the wound of lower extremities caused by skin infection in elderly patients. J Tissue Eng Reconstr Surg. (2020) 16(5):374–7. 10.3969/j.issn.1673-0364.2020.05.006

[15] He S. Effect of vacuum sealing drainage technique combined with thickness auto skin grafting in the treatment of hand hot crash injury. J Trauma Surg. (2018) 20(01):58–60. 10.3969/j.issn.1009-4237.2018.01.015

[16] Liu JC Wang LN Chen FS Zhang GT. Experience of application of skin flap in repairing 112 patients with severe thermal pressure injury of hand. Chin J Reparative Reconstr Surg. (2000) 14(4):197–9. doi: CNKI:SUN:ZXCW.0.2000-04-001 12078299

[17] Lin WH Zheng TZ Wang QS Pang SF. Study on the effect of pedicle skin flap or subdermal vascular plexus on repairing the hand injury. Chin J Reparative Reconstr Surg. (2005) 07:528–30. doi: CNKI:SUN:ZXCW.0.2005-07-011 16108335

[18] Lv GZ Huang YS. National experts consensus on clinical application of collagen-based wound biomaterials (2018 version). Chin J Damage Repair (Electronic Edition). (2018) 13(06):406–9. doi: CNKI:SUN:GRYX.0.2018-04-002

[19] Ba T Wang LF Cao SJ Xinba YE. Application of abdominal thin skin flap to repair 26 cases of hand thermal compression injury. Chin J Burns. (2004) 20(2):1. 10.3760/cma.j.issn.1009-2587.2004.02.023.

[20] Li JL Wang YC Xue CY Li JH Zhu J. Artificial dermis combined with KCI negative pressure suction device in repair of skull exposure after scalp malignant tumor surgery. Chin J Med Aesthetics Beauty. (2021) 27(3):199–202. 10.3760/cma.j.issn.1671-0290.2021.03.011

[21] Geng LL Zeng D Tao BJ Lv GP Yan H Yang HM Treatment of a case of refractory hip wound complicated with chronic osteomyelitis. Chin J Damage Repair (Electronic Edition). (2021) 16(3):273–5. 10.3877/cma.j.issn.1673-9450.2021.03.019

[22] Morykwas MJ Argenta LC Shelton-Brown EI McGuirt W. Vacuum-assisted closure: a new method for wound control and treatment: animal studies and basic foundation. Ann Plast Surg. (1997) 38(6):553–62. 10.1097/00000637-199706000-00001 9188970

[23] Liu T Qiu C Ben C Li H Zhu S. One-step approach for full-thickness skin defect reconstruction in rats using minced split-thickness skin grafts with Pelnac overlay. Burns Trauma. (2019) 7(1):19. 10.1186/s41038-019-0157-0 31413962 PMC6691548

[24] Jiang K Ding YH Ding XH. Clinical application of bilayer artificial dermis for repair of fingertips and nail bed defects. Chin J Hand Surg. (2020) 36(3):203–5. 10.3760/cma.j.cn311653-20200223-00062

[25] Yu ZY Zhang XL Liu XH Li X Qin Y Geng SL. Application of Lando double layer artificial dermis in hand skin defect. J Pract Hand Surg. (2021) 35(1):13–5. 10.3969/j.issn.1671-2722.2021.01.004

[26] Heimbach DM Warden GD Luterman A Jordan MH Ozobia N Ryan CM Multicenter postapproval clinical trial of Integra((R)) dermal regeneration template for burn treatment. J Burn Care Rehabil. (2003) 24(1):42–8. 10.1097/00004630-200301000-00009 12543990

[27] Tufaro AP Buck DW Fischer AC. The use of artificial dermis in the reconstruction of oncologic surgical defects. Plast Reconstr Surg. (2007) 120(3):638–46. 10.1097/01.prs.0000270298.68331.8a 17700115

[28] Li DB Zhang ZJ Tian HJ. One patient with chronic refractory wound caused by barnboo sticks in lower leg. Chin J Burns. (2018) 34(10):727–8. 10.3760/cma.j.issn.1009-2587.2018.10.014 30369142

[29] Yeong E-K Chen S-H Tang Y-B. The treatment of bone exposure in burns by using artificial dermis. Ann Plast Surg. (2012) 69(6):607–10. 10.1097/SAP.0b013e318273f845 23154329

[30] Yang L Pan SX Xue JR Jiang P Guo XZ. Effect of artificial dermis combined with autologous skin transplantation on wound surface repair. Chin J Modern Operative Surg. (2017) 21(02):141–4. 10.16260/j.cnki.1009-2188.2017.02.015

[31] Liu K Sun YZ Zhao YB Zhao YB Tian LJ Xu Y Comparison of therapeutic effects between artificial dermis and skin flap repair in the treatment of open lower limb injuries. Chin J Bone Joint Injury. (2019) 34(6):657–8. 10.7531/j.issn.1672-9935.2019.06.035

[32] Wang F Zhou P Duan SF Gong YZ Xu ZD Chen X. Application of artificial dermis combined with vacuum sealing drainage with instillation in tendon or bone exposure wounds repair. Chin J Damage Repair (Electronic Edition). (2020) 15(06):470–4. 10.3877/cma.j.issn.1673-9450.2020.06.008

[33] Sobti N Ji E Brown RL Cetrulo CL Jr Colwell AS Winograd JM Evaluation of acellular dermal matrix efficacy in prosthesis-based breast reconstruction. Plast Reconstr Surg. (2018) 141(3):541–9. 10.1097/PRS.0000000000004109 29481386

